# Behavioural evidence for self-medication in bumblebees?

**DOI:** 10.12688/f1000research.6262.3

**Published:** 2015-10-29

**Authors:** David Baracchi, Mark J. F. Brown, Lars Chittka

**Affiliations:** 1 Department of Biological and Experimental Psychology, School of Biological and Chemical Sciences, Queen Mary University of London, Mile End Road, London, E1 4NS, UK; 2 School of Biological Sciences, Royal Holloway University of London, Egham, Surrey, TW20 0EX, UK

**Keywords:** Bombus terrestris, Crithidia bombi, foraging, nicotine, pathogens, pollinators, pollinator-plant interactions, secondary metabolites

## Abstract

The presence of antimicrobial secondary metabolites in nectar suggests that pollinators, which are threatened globally by emergent disease, may benefit from the consumption of nectars rich in these metabolites. We tested whether nicotine, a nectar secondary metabolite common in *Solanaceae *and *Tilia* species, is used by parasitized bumblebees as a source of self-medication *, *using a series of toxicological, microbiological and behavioural experiments. Caged bees infected with *Crithidia bombi* had a slight preference for sucrose solution laced with the alkaloid and behavioural tests showed that the parasite infection induced an increased consumption of nicotine during foraging activity, though nicotine had an appetite-reducing effect overall. When ingested, nicotine delayed the progression of a gut infection in bumblebees by a few days, but dietary nicotine did not clear the infection, and after 10 days the parasite load approached that of control bees. Moreover, when pathogens were exposed to the alkaloid prior to host ingestion, the protozoan’s viability was not directly affected, suggesting that anti-parasite effects were relatively weak. Nicotine consumption in a single dose did not impose any cost even in starved bees but the alkaloid had detrimental effects on healthy bees if consistently consumed for weeks. These toxic effects disappeared in infected bees, suggesting that detoxification costs might have been counterbalanced by the advantages in slowing the progression of the infection. Nicotine consumption did not affect bee lifespan but the reduction in the parasite load may have other likely unexplored subtle benefits both for individual bees and their colony.  Potential evidence for self-medication is discussed. The contention that secondary metabolites in nectar may be under selection from pollinators, or used by plants to enhance their own reproductive success, remains to be confirmed.

## Introduction

Parasites can have a dramatic impact on their hosts, and consequently provide a powerful selective force for host defence mechanisms. Molecular mechanisms (e.g. the innate and adaptive immune system) are traditionally considered the major anti-parasite defences in the animal kingdom. However, hosts can rely on a range of alternative defence mechanisms, such as morphological barriers ( St Leger, 1991), changes in life-history traits ( Michalakis, 2009), symbiont-mediated defences ( Oliver *et al.*, 2010) and altered behaviours ( de Roode & Lefèvre, 2012; Moore, 2002).

Behavioural immunity is an important modality of defence against diseases ( de Roode & Lefèvre, 2012), and medication behaviour is a key immune mechanism in some animals ( Clayton & Wolfe, 1993; de Roode *et al.*, 2013). Medication behaviour has been defined as the selective use of anti-pathogenic substances by infected individuals ( Lozano, 1998; Singer *et al.*, 2009), with a measurable benefit to host fitness and negative effects on the pathogen ( Abbott, 2014; Clayton & Wolfe, 1993; Singer *et al.*, 2009). As an additional criterion to support the notion that the substance is only of value as medication, it has been proposed that healthy individuals must suffer a cost when consuming it ( Abbott, 2014).

Evidence for self-medication in animals comes from the consumption of curative plants by vertebrates ( Rodriguez & Wrangham, 1993). Many plants contain metabolites that display a wide range of biological activities ( Cowan, 1999) which were originally evolved to combat herbivores or plant-parasites ( Hadacek, 2002). Chimpanzees, *Pan troglodytes,* modify their diet to include particular plant species containing medicinal substances to cure helminth infections ( Mooney & Agrawal, 2008; Wrangham, 1995). There are also experimental explorations of self-medication in insects, for example two species of woolly bear caterpillars, which increase their preference for pyrrolizidine alkaloids or iridoid glycosides when parasitized, improving their chances of surviving parasitoid infection ( Bernays & Singer, 2005; Singer *et al.*, 2009; Smilanich *et al.*, 2011). Similarly, fruit fly larvae infected by parasitoid wasp larvae preferentially consumed high-ethanol fly food as a medicine, again increasing their survival ( Milan *et al.*, 2012), while no evidence for self-medication to nematode parasitism has been found in the fly *Drosophila putrida* ( Debban & Dyer, 2013). Trans-generational medication, but not self-medication, has been described in the monarch butterfly ( Lefevre *et al.*, 2010) and self-medication has been hypothesized for honeybees that increase plant resin collection in response to a fungal infection ( Simone-Finstrom & Spivak, 2012). Finally, ants apply antimicrobial venomous secretion to the cuticle of contaminated larvae to medicate their brood ( Tragust *et al.*, 2013).

Animal societies arguably face intense pressure from pathogens, because of the high number of individuals living in high densities, relatively low genetic variability, and the relatively constant, high levels of humidity and warm temperatures within their nests ( Schmid-Hempel, 1998). In addition, social pollinators, such as bumblebees and honeybees, are often exposed to an increased risk of infection via flowers (reviewed in McArt *et al.*, 2014), which represent a shared “public place” where homo- and hetero-colonial conspecifics and other heterospecific pollinators feed repeatedly every day. Given the potential importance of parasites and disease in driving declines of managed honeybees ( de Miranda & Genersch, 2010; Rosenkranz *et al.*, 2010) and wild bumblebees ( Cameron *et al.*, 2011; Fürst *et al.*, 2014; Schmid-Hempel *et al.*, 2014; McMahon *et al.*, 2015), understanding the potential relevance of pharmacophagy to social pollinators may be a key to understanding and managing these declines.

Here we use an important natural and managed pollinator, the bumblebee *Bombus terrestris*, and its parasite *Crithidia bombi* to investigate the potential for pharmacophagy in social pollinators. *C. bombi*, a trypanosome gut parasite, is the most prevalent parasite of bumblebees ( Shykoff & Schmid-Hempel, 1991). The parasite, transmitted either vertically or horizontally ( Durrer & Schmid-Hempel, 1994; Otterstatter & Thomson, 2007), infects adults *per os,* and two-three days post infection, infective cells are released through the faeces of bees ( Schmid-Hempel & Schmid-Hempel, 1993). Queens infected by *C. bombi* have a reduced success in colony founding ( Brown *et al.*, 2003), and produce fewer reproductive offspring ( Brown *et al.*, 2003), while infected workers experience a higher mortality rate under stressful conditions ( Brown *et al.*, 2000). Moreover, infection impairs foraging success and learning abilities, inducing additional costs to the colony ( Alghamdi *et al.*, 2008; Gegear *et al.*, 2006). Recent research ( Manson *et al.*, 2010; Richardson *et al.*, 2015) has shown that several secondary metabolites such as alkaloids (including nicotine) and glycosides, reduce the *C. bombi* load after consumption by the bumblebee species *Bombus impatiens*, suggesting that these pollinators might exploit nectar toxins or other metabolites to self-medicate.

To test whether bumblebees are able to self-medicate using naturally occurring nectar secondary metabolites we conducted a series of toxicological, microbiological and behavioural experiments using a different species of *Bombus* ( *B. terrestris*) and *C. bombi* as models and nicotine as a natural nectar alkaloid. Nicotine is encountered by pollinators at variable concentrations between 0.1 ng/μl and 3 ng/μl in floral nectar of *Nicotiana* species (native of South America and naturalised worldwide by humans) and *Tilia* species (native in most of the temperate Northern Hemisphere) ( Detzel & Wink, 1993; Naef *et al.*, 2004; Tadmor-Melamed *et al.*, 2004).

## Methods

### Insects, pathogens and compounds

All experiments were performed with worker bumblebees ( *B. terrestris*) obtained from a continuous rearing program (provided by Koppert B.V., The Netherlands) and conducted under standardized laboratory conditions. The insects were provided *ad libitum* with commercial pollen (provided by Koppert B.V., The Netherlands) and 30% sucrose solution as protein source and energy respectively. The parasites (the protozoan flagellates *C. bombi*) that we used for the experimental infections stemmed from several naturally infected colonies that were laboratory-raised from infected queens. (-)-Nicotine hemisulphate salt (≥95% (TLC), ~40% (w/v) in H _2_O (based on free base); N1019 Sigma) was used in all experiments.

### Infection experiments

To determine whether the nectar alkaloid nicotine influences the severity of *C. bombi* infections in bumblebees, we designed two experiments following Manson *et al.* (2010). In the “Continuous Exposure” test, subjects were first inoculated with *C. bombi* and subsequently fed on a daily supply of nicotine solution or sucrose solution (Control), mimicking the continuous exposure to nectar constituents by a bumblebee worker. In the “Delayed Exposure” test, we first exposed directly *C. bombi* cells to nicotine or control solutions for two hours before inoculating bees, and then we fed them on a sucrose-only solution. We subsequently compared the parasite load in inoculated bumblebees.

We collected faeces from 30 workers from three infected colonies, in order to generate a mix of different parasite strains. The faeces were mixed for one minute with a vortex mixer and the *C. bombi* cocktail was left to stand at room temperature for two hours. Following this, the supernatant was removed and thoroughly mixed. Cell counts were made using a haemocytometer. Following Manson *et al.* (2010), faeces were mixed with sucrose solution, generating an inoculum concentration of 2,000 parasite cells/μl. Before inoculation, bees were not given any nutrition for two hours to facilitate infection. Bees derived from two different healthy colonies were screened to ensure that they were free of parasites. Bees were individually presented with a 10 μl droplet of inoculum. We observed foragers until the inoculum was consumed in its entirety. Each bee thus ingested approximately 20,000 parasite cells. This value is within the range of *C. bombi* cells present in the faeces of infected workers ( Logan *et al.*, 2005), thus mimicking a realistic infection level for transmission to healthy bees.

Post inoculation, in the “Continuous Exposure” test, bees from three colonies were kept individually in Petri dishes and either given a 0.5 ml solution of 2.5 ng/μl nicotine (nectar concentration in the natural range of this alkaloid) in 30% sucrose solution (Experimental bees, *n* = 20) or 0.5 ml of plain 30% sucrose solution (Control bees, *n* = 20) every day for 10 days. All bees were given a 1g pollen ball every day. In the “Delayed Exposure” test, the *C. bombi* inoculum was exposed to nicotine in the dark for two hours before the mixture was offered to bees for ingestion. This mimics a direct exposure of the pathogen to nicotine-laced nectar in a flower. C. *bombi* cells were placed in a solution of 2.5 ng/μl nicotine in 30% sucrose (Experimental treatment), and in a solution of 30% sucrose only (Control treatment). Two hours later, 20 Experimental bees and 20 Control bees were inoculated (for inoculum preparation see above). The treatment emulates a situation where *Crithidia* cells are deposited on a flower by infected bees and the flower is then visited by a healthy bee. Post inoculation, bees of both groups were kept individually in Petri dishes. They were provided with a fresh pollen ball and 0.5 ml of 30% sucrose solution every day.

Infection levels were determined 7 and 10 days after inoculation (the period of time in which parasite load peaks and plateaus ( Schmid-Hempel & Schmid-Hempel, 1993)). Each bee was removed from its Petri dish and put into a small glass tube until it defecated. In some individual bees, too little rectal fluid was available after the initial screen; in such cases, we repeated the procedure some hours later. Faeces were transferred to a haemocytometer to count the number of parasite cells.

### Laboratory toxicity bioassays

In order to determine the impact of nicotine consumption on bumblebee survival and any possible interactive effects of dietary toxin consumption and physiological stress (for which we used starvation, as *Crithidia* has its biggest detrimental impacts on starved bees ( Brown *et al.*, 2000)), we exposed bumblebees to artificial nectars with or without nicotine, and then kept them under starvation or with *ad libitum* food conditions. “Starved bees” were moved individually from their nest into Petri dishes, starved for two hours and fed either with *ad libitum* 30% sucrose solution food for 30 minutes (Starved, Control) or 2.5 ng/μl nicotine in 30% sucrose (Starved, Nicotine). Survival censuses were conducted every hour until all bees were dead. “ *Ad libitum* food bees” were kept individually in Petri dishes, and provided, every day, with 0.5 ml of 30% sucrose solution plus a fresh pollen ball (Control *ad libitum* food), 2.5 ng/μl nicotine in 30% sucrose solution and, again, a fresh pollen ball (Nicotine *ad libitum* food), 2.5 ng/μl nicotine in 30% sucrose solution on day 0 and 0.5 ml of 30% sucrose solution (Nicotine-once *ad libitum* food) and a fresh pollen ball on a daily basis ( Manson *et al.*, 2010). Survival censuses were conducted daily until all bees had died. For each of the five treatments we chose bees from three different young healthy colonies and we randomised bees across treatment groups. Each treatment group was composed of 60 bees (20 bees per colony). Comparisons of the survival parameters of bumblebees in all treatments allowed us to evaluate the effect of nicotine, starvation, and colony membership on survival. We checked for dead bees twice daily and thus such individuals could be weighed within 12h of their death using a microscale (Navigator N30330, Ohaus, Pine Brook, USA).

### Trade-off between detrimental and beneficial effects of nicotine

To evaluate whether infected bees benefit from the consumption of nicotine in terms of survival and/or parasite load, we conducted two additional experiments in which infected bumblebees received artificial nectars enriched with nicotine or not and were maintained either starved (three groups of 30 bees, 10 bees from three different colonies, 90 bees in total) or provided with *ad libitum* food (three groups of 45 bees, 15 bees from three different colonies, 135 bees in total). In both experiments the three groups of bees were inoculated with *C. bombi* as described above and individually kept in Petri dishes under three types of diet (each diet consisted of two solutions dispensed by two different Eppendorf tubes): Control Group: 30% sucrose only in both dispensers (Suc-Suc group); Exp. Group 1: 2.5 ng/μl nicotine in 30% sucrose in both dispensers (Nic-Nic group); Exp. Group 2: 30% sucrose only in one dispenser and 2.5 ng/μl nicotine in 30% sucrose in the other one (Suc-Nic group). “Starved bees” were fed for 12 days and then starved until all bees were dead. The infection levels were checked on days 7 and 10 after inoculation. Survival censuses were conducted every hour (starved bees) and every day ( *ad libitum* food bees) until all bees were dead. At the end of the experiment we quantified total consumption of artificial nectars in each dispenser for each bee. Comparison of the survival parameters of bumblebees in all treatments enabled us to quantify the effect of nicotine and starvation on survival.

### Behavioural test

For testing, each bee colony was housed in a wooden nest box (28 × 20 × 11 cm) connected to a wooden flight arena with a transparent, UV-transmitting Plexiglas lid (120 × 100 × 35 cm), by means of a transparent Plexiglas tube. Shutters along the length of this tube enabled control of the traffic of bees between nest boxes and flight arena ( Chittka, 1998). Each bumblebee was individually marked with a coloured numbered disk.

Bees were pre-trained to forage on 12 square transparent plastic flowers of 24 × 24 mm (Perspex ^®^ Neutral) organized in two patches equidistant from the entrance of the nest. Plastic chips were placed on vertical transparent glass cylinders to raise them above the green floor of the flight arena. During the pre-training all flowers were rewarding with a 15 μl droplet of 30% sucrose solution, placed in a well in the centre of the flower ( Raine & Chittka, 2008). This provided bees with an equal chance to associate both these patches (left and right) with reward during the pre-training period. Bees were allowed to forage freely on these flowers which were refilled as soon as the bees moved on a different artificial flower. In this way bees never experienced an empty flower with the exception of the last visited one. The number of foraging trips (bouts) made in the flight arena by each bee was observed to ensure only strongly motivated foragers visiting both patches (bees that did at least five consecutive foraging bouts) were selected for the experiment ( Raine *et al.*, 2006).

After pre-training, the preference of both healthy and infected pre-trained bees was tested for blue plastic flowers (Perspex ^®^ 727) containing nicotine (one patch reward: 2.5 ng/μl nicotine in 30% sucrose solution; one patch reward: only 30% sucrose solution). Each bee ( *n* = 31 infected bees; *n* = 28 healthy bees) was tested individually and one hundred consecutive choices were recorded after the first bout was initiated. Bees were regarded as choosing a flower when they landed and fed from it. Bees approaching or just briefly touching a flower were not considered as choosing that flower. As in the pre-training, flowers were refilled after the bee moved to a different one so that bees never experienced an empty flower with the exception of the last visited one. Flowers were washed between subsequent bees in order to remove possible scent marks ( Saleh & Chittka, 2006). The patch formed by nicotine-containing flowers was swapped from left to right for half the bees of each group (healthy and infected bees). Controlled illumination was provided by high frequency fluorescent lighting [(TMS 24F) lamp with HF-B 236 TLD (4.3 Khz) ballasts, Phillips, Netherlands fitted with Activa daylight fluorescent tubes, Osram] which simulated natural daylight ( Dyer & Chittka, 2004). At the end of the experiment all the bees were sacrificed and the concentration of *C. bombi* in their hind gut was determined (see above).

### Statistical analysis

In the infection experiments, 10 out of 80 bees perished by day 10 for unknown causes. Thus, we quantified the infection intensities of 40 (day 7) and 36 (day 10) bees in the “Continuous Exposure” experiment, and 37 (day 7) and 34 (day 10) bees in the “Delayed Exposure” experiment. To compare differences in parasite load between control and experimental bees 7 and 10 days after inoculation in both experiments we used a generalized linear mixed model (GLMM), with pathogen counts as the within-subject variable and *C. bombi* exposure to nicotine, time (day 7 and day 10), colony of origin, and bee body weight as explanatory factors. As the data were not normally distributed and homogeneity of variances and sphericity could not be assumed in several cases, we performed corrections according to Huynh-Feldt epsilon ( Field, 2009). For the statistical evaluations in the survival experiments, we used the classical survival parameters (i.e. the survival distribution and the median survival time (LT50)). The survival distributions for all treatments were quantified using the Breslow Statistic (Mantel–Cox Test). The following variables were entered in the regression model: colony of origin, body weight, nicotine treatment. For the behavioural experiment, a T test was used to examine differences between preferences for nicotine-rich nectar and control nectar in healthy and infected bees. Spearman rank correlation tests were used to correlate parasite load and nicotine preference. All statistical analyses were done on SPSS 13 ^®^ for Windows.

## Results

### Infection experiments

**Figure 1.**
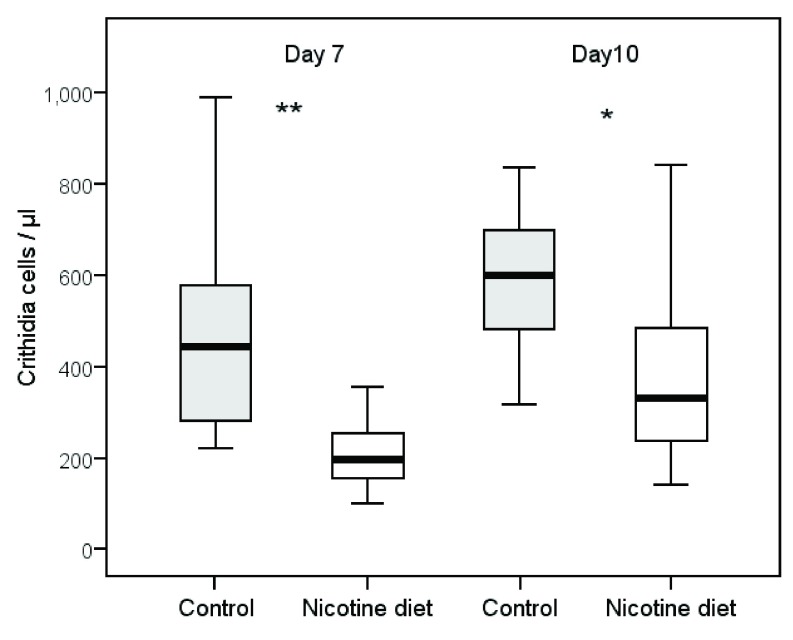
Intensity of *C. bombi* infections in bumblebees that received either a nicotine diet (Experimental bees, *n* = 20) or a sucrose only diet (Control bees, *n* = 20). Faeces were checked after 7 days and 10 days post inoculation. Box plots show medians, 25 ^th^ and 75 ^th^ percentiles (** *P* < 0.001; * *P* = 0.001).

In both the “Continuous Exposure” and the “Delayed Exposure” tests control bees had comparable levels of *C. bombi* infection (t test, day 7: *t* = 0.16, df = 37, *P* = 0.98; day 10: *t* = 0.92, df = 34, *P* = 0.36). In the “Continuous Exposure” test, a diet laced with nicotine reduced the intensity of *C. bombi* infections in bee workers ( Dataset 1). GLMM analysis revealed significant main effects of nicotine and time since inoculation on infection intensity, but not colony of origin or bee body weight ( Table 1). At both 7 and 10 days after inoculation, bees exposed to nicotine had infections that were (on average) 1.11 and 0.56 times respectively less intense than control bees (t test, day 7: *n* = 20-20, *t* = 5.2, df = 38, *P* < 0.001; day 10: *n* = 18-18, *t* = 3.47, *df* = 34, *P* = 0.001; Figure 1). Infection intensity increased significantly from day 7 to day 10, independently of nicotine treatment (no-significant Nicotine and Colony x Time effect; Table 1).

**Table 1.** “Continuous Exposure” test: results from GLMM analysis of *C. bombi* population dynamics in bumblebees.

*Factor*	*F-value*	*Df*	*P-value*
Nicotine diet	35.3	1,61	0.001
Time since inoculation	16.2	1,61	0.001
Bee body weight	1.07	1,61	0.3
Colony	0.46	1,65	0.8
Interactions	-	-	N.S.

**Figure 2.**
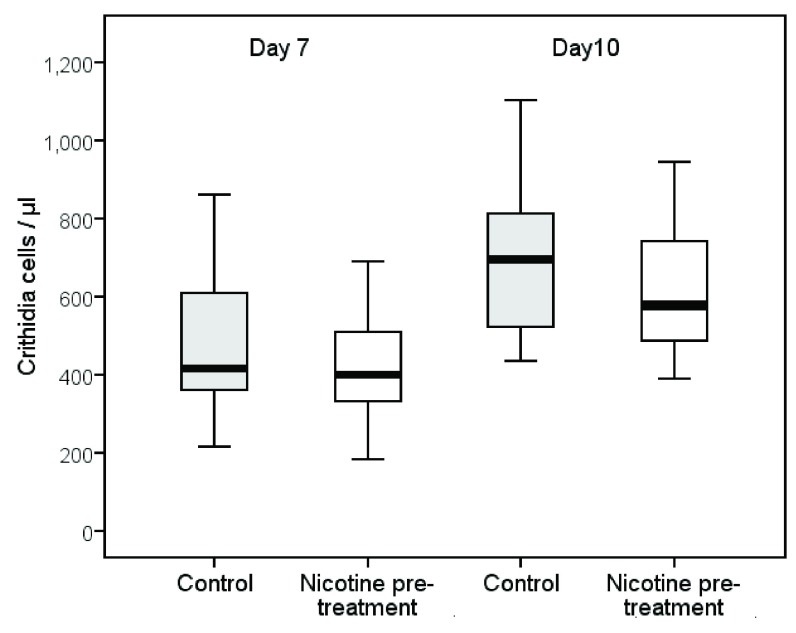
Intensity of *C. bombi* infections in bumblebees inoculated with pathogens previously exposed to nicotine for two hours (Experimental bees, *n* = 20) or to a control sucrose diet (Control bees, *n* = 20). Faeces were checked after 7 days and 10 days post inoculation. Box plots show medians, 25 ^th^ and 75 ^th^ percentiles ( *P* = N.S.).

In the “Delayed Exposure” test, exposing *C. bombi* to nicotine for two hours before inoculation had no effect on parasite load ( Table 2) ( Dataset 1). At 7 days and 10 days post-inoculation, bees exposed to nicotine had infections that on average were as intense as those of control bees (t test, day 7: *n* = 19-18, *t* = 0.16, df = 35, *P* = 0.87; day 10: *n* = 17-17, *t* = -0.69, df = 32, *P* = 0.5; Figure 2). Infections intensified significantly from day 7 to day 10, independently of nicotine treatment (there was no significant Nicotine x Time and Colony x Time effects; Table 2). Taken together, these findings prove the antimicrobial activity of nicotine against the pathogen when ingested by bumblebees, but also indicate that when pathogens are exposed to the alkaloid prior to host ingestion the protozoan’s viability is not strongly affected.

**Table 2.** Delayed Exposure test: results from the GLMM analysis of *C. bombi* population dynamics in bumblebees.

*Factor*	*F-value*	*Df*	*P-value*
Nicotine pre-treatment	0.02	1,62	0.8
Time since inoculation	27.1	1,60	0.001
Bee body weight	0.52	1,62	0.4
Colony	2.9	1,62	0.1
Interactions	-	-	N.S.

Infection experiments Effect of nicotine on parasite load in infected bumblebees. Click here for additional data file. Copyright: © 2015 Baracchi D et al. 2015 Data associated with the article are available under the terms of the Creative Commons Zero "No rights reserved" data waiver (CC0 1.0 Public domain dedication).

### Laboratory toxicity bioassays

**Figure 3.**
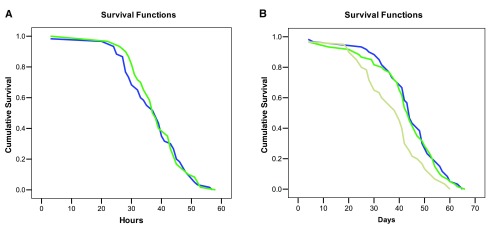
**A**: Cumulative survival of healthy bees fed with a sucrose solution with (blue line) or without (green line) nicotine and starved. **B**: Cumulative survival of healthy bees that received a daily diet of sucrose solution with (beige line), or without nicotine (blue line), or a single dose of nicotine on day one (green line).

In the “Starved” test, statistical evaluation of the survivorship of control and experimental bumblebees revealed that a nicotine diet was not a significant predictor of mortality (Log-rank Mantel Cox test χ ^2^ = 0.21, df = 1, *P* = 0.88; Figure 3A) ( Dataset 2). Furthermore no effect of colony of origin and bee body weight on mortality was found (GLM, treatments: F = 1.1, df = 1, *P* = 0.29; Colony F = 0.46, df = 2, *P* = 0.63; body weight: F = 0.19, df = 1, *P* = 0.66). The median lethal time (LT50) for the two groups did not differ (control LT50: 39 hours, exp. bees LT50 = 37 hours).

In the “ *ad libitum* food” test a Log-rank Mantel Cox test showed that a daily diet including nicotine was a significant predictor of mortality (χ ^2^ = 11.56, df = 2, *n* = 180, *P* = 0.003; Figure 3B) ( Dataset 2). Pairwise statistical comparisons revealed that bees fed consistently with nicotine had significantly lower survivorship than ‘Nicotine-once’ and ‘Control bumblebees’ ( *P* = 0.001), while the latter two experimental groups did not differ ( *P* = 0.86). LT50 of bees fed daily with nicotine was 39 days while ‘Nicotine-once’ bumblebees and control bees had a LT50 of 44 and 43 days respectively. Colony of origin and body weight did not affect bee mortality (GLM, Colony: F = 0.35, df = 2, *P* = 0.71; body weight: F = 1.90, df = 1, *P* = 0.16), but we found a significant interaction between body weight and treatment (larger bees were less susceptible to nicotine, GLM, F = 5.12, df = 1, *P* = 0.025). Taken together, these findings indicate that nicotine has some detrimental effects on healthy bumblebees if consistently consumed for weeks but also that these effects are possibly quite weak.

Revision 1. Laboratory toxicity bioassays Effect of nicotine on healthy bee survival. The ‘Body weight (ad libitum)’ column previously contained duplicate data, the correct values have now been reinstated. Click here for additional data file. Copyright: © 2015 Baracchi D et al. 2015 Data associated with the article are available under the terms of the Creative Commons Zero "No rights reserved" data waiver (CC0 1.0 Public domain dedication).

### Trade-off between detrimental and beneficial effects of nicotine

In both “ *ad libitum* food bees” and “starved bees” tests, a nicotine diet was not a significant predictor of survival (Log-rank Mantel Cox test: “ *ad libitum* food bees”: *n* = 135, Nic-Nic vs Nic-Suc χ ^2^ = 0.3, *P* = 0.6; Nic-Nic vs Suc-Suc χ ^2^ = 0.01, *P* = 0.9; Nic-Suc vs Suc-Suc χ ^2^ = 0.7, *P* = 0.4; “Starved bees”, *n* = 76; Nic-Nic vs Nic-Suc χ ^2^ = 0.4, *P* = 0.5; Nic-Nic vs Suc-Suc χ ^2^ = 0.1, *P* = 0.7; Nic-Suc vs Suc-Suc χ ^2^ = 0.01, *P* = 0.9) ( Dataset 3). Furthermore no effect of colony of origin on mortality was found (GLM, “ *ad libitum* food bees”: F = 1.4, df = 2, *P* = 0.24; “Starved bees”: GLM, F = 2.02, df = 2, *P* = 0.14). The median lethal time LT50 for the three groups did not differ (“ *ad libitum* food bees”: Suc-Suc LT50: 22 days, Nic-Suc LT50 = 23, days, Nic-Nic LT50 = 22; “Starved bees”: Suc-Suc LT50: 25 hours, Nic-Suc LT50 = 28 hours, Nic-Nic LT50 = 31 hours).

GLMM analysis revealed significant main effects of treatment (df = 2, F = 3.46, *P* = 0.03) and time since inoculation (df = 1, F = 57.3, *P* < 0.001) on infection intensity, but not colony of origin (df = 2, F = 1.64, *P* = 1.96). No interaction between diet, time and colony was significant. Overall bees caged in Petri dishes consumed less food over the entire duration of the experiment if exposed to nicotine (Anova test: F = 9.68, *n* = 90, df = 2, 87, *P* = 0.001; Dunnett T3 post hoc test: Suc-Suc vs Nic-Nic and Suc-Suc vs Nic-Suc *P* < 0.001) ( Dataset 4). Infected bees showed a slight preference (54 ± 17%) for sucrose solution laced with nicotine (Paired samples t test, t = 2.14, df = 29, *n* = 30, *P* = 0.04).

Overall these findings indicate that, even though nicotine reduces the parasite load in infected bees, and such bees have a slight preference for sucrose solution laced with the alkaloid, there is no net benefit in term of survival for infected bees.

Trade-off between detrimental and beneficial effects of nicotine Dietary nicotine effect on parasite load and life expectancy in infected bumblebees. Click here for additional data file. Copyright: © 2015 Baracchi D et al. 2015 Data associated with the article are available under the terms of the Creative Commons Zero "No rights reserved" data waiver (CC0 1.0 Public domain dedication).

Diet preference of caged bees Caged infected bee preference for nicotine-laced nectars. Click here for additional data file. Copyright: © 2015 Baracchi D et al. 2015 Data associated with the article are available under the terms of the Creative Commons Zero "No rights reserved" data waiver (CC0 1.0 Public domain dedication).

### Preference of freely flying bees for nicotine-laced flowers

**Figure 4.**
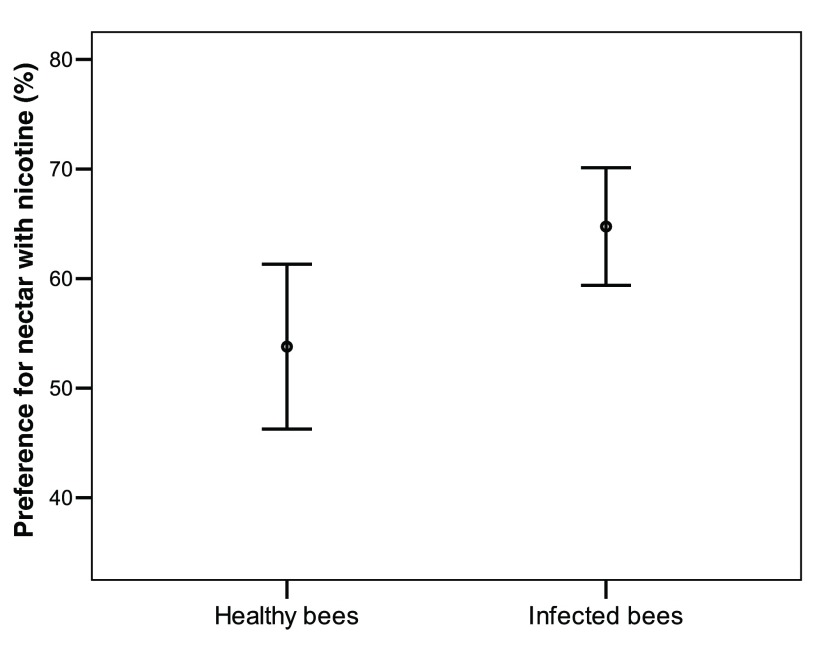
Percentage of preferred flowers rewording with nicotine-rich artificial nectar by infected bees ( *n* = 31) and healthy bees ( *n* = 28), (t test, *P* = 0.016). Infected bees visited nicotine-containing flowers 64.5 ± 13.8 (s.d.) times while healthy bees visited them 54.8 ± 19.4 (s.d.) times.

Infected bumblebees allowed to forage on plastic flowers showed a significantly increased propensity to visit nicotine rewarding flowers when compared to healthy bees (t test, *n* = 31, 28, t = -2.4, df = 57, *P* = 0.016; Figure 4) ( Dataset 5). Indeed on 100 consecutive choices infected bees visited the nicotine flowers on average 64.5 ± 13.8 (s.d.) times while healthy bees visited them 54.8 ± 19.4 (s.d.) times. Since test bees were introducing nicotine into the colony throughout testing, we controlled for prior exposure to nicotine effect on nicotine preference. Bees tested later in the experiment did not show a higher or lower nicotine preference (Spearman test, Infected bees: ρ = -0.21, *n* = 31, *P* = 0.3; Control bees *n* = 28, ρ = 0.041, *P* = 0.8). There was no correlation between pathogen load and the propensity of infected bees to visit flowers with nicotine-rich artificial nectar (Spearman test: *n* = 31, ρ = 0.19, df = 29, *P* = 0.28).

Behavioural test Percentage of preferred flowers rewording with nicotine-rich artificial nectar by infected and healthy bees. Click here for additional data file. Copyright: © 2015 Baracchi D et al. 2015 Data associated with the article are available under the terms of the Creative Commons Zero "No rights reserved" data waiver (CC0 1.0 Public domain dedication).

## Discussion

Here we demonstrate that parasitized bumblebees modify their diet preference and foraging behaviour, delaying the development of an infection. In our experimental setup the parasite infection induced an increased consumption of nicotine both in individually caged as well as in foraging bumblebees. Healthy bees exposed to nicotine suffer an appreciable cost, also in line with the key criteria for self-medication ( Abbott, 2014; Clayton & Wolfe, 1993; Singer *et al.*, 2009). However, despite this preferential ingestion of a “non-nutritive” antimicrobial alkaloid by infected bees, this behaviour appears to be of limited efficiency since dietary nicotine does not fully cure *C. bombi* infection, or increase longevity of infected bees, similarly to a recent study on the North American *Bombus impatiens* ( Richardson *et al.*, 2015). Nonetheless bumblebees in our study exhibited a reduced *C. bombi* load after daily consumption of the alkaloid making the existence of a potential self-medication phenomenon plausible. In nature, infection entails an array of costs and higher mortality under stressful conditions ( Alghamdi *et al.*, 2008; Brown *et al.*, 2000; Brown *et al.*, 2003; Gegear *et al.*, 2006). As a consequence, any reduction in the severity or progression of infection in bees, induced by mechanisms such as the consumption of nectar containing curative alkaloids (e.g. gelsemine ( Manson *et al.*, 2010), anabasine and nicotine ( Richardson *et al.*, 2015)), might be beneficial in terms of fitness for both bees and colonies.

Even if we cannot completely exclude that the limited effect of nicotine is due to the initial challenge being too strong for the nicotine to have a measurable influence on life expectancy, both nicotine concentration and *Crithidia* inocula used in our study simulated natural doses. It is, however, possible that the nicotine concentration available to workers within the colony is substantially different from that found in flowers. At one end of the scale of possibilities, nicotine-laced nectar might be regurgitated into honeypots containing nicotine-free nectar, resulting in further dilution of the nicotine concentration and even lower anti-microbial effects. On the other hand, if some individual foragers that have discovered alkaloid-containing nectar deposit it predominantly into certain honeypots, and concentration is further increased by evaporation and possibly modified by enzymatic addition, then such honeypots could contain substantially higher nicotine concentrations than found in floral nectar ( Thomson, 2015). It is possible that such honeypots could be a kind of colony ‘pharmacy’ specifically used for self-medication of infected workers, or to feed larvae to limit the spread of an infection within the colony (James Thomson, personal communication). Similar considerations apply to pollen, which might also contain alkaloids with antimicrobial properties ( Thomson, 2015). Additional field and mesocosm tests, as well measurements of alkaloid concentration in colony honeypots are thus needed to evaluate the actual concentrations to which different colony members and the brood are exposed, and thus to clarify the actual benefits of differential foraging for such substances.

Nicotine also has a costly effect on uninfected individuals, as shown by our toxicological assays. A daily diet containing nicotine, lasting more than two months, reduced the life expectancy of bumblebees, and this effect was stronger in smaller bees. This might possibly be aggravated in the wild, where bees are exposed to other stressors and do not have access to *ad libitum* food. However, we note that differences in mortality rate between controls and nicotine-treated bees started to be evident only after 20 days from the first exposure suggesting that in nature this detrimental effect may be mitigated due to the relatively short lifespan of foragers in the wild ( da Silva-Matos & Garófalo, 2000). Moreover, in nature, bees are unlikely to visit a single nectar source continuously for weeks as in our experiments, further reducing the negative effect of nicotine intake. In infected bumblebees, which show a shorter lifespan than healthy ones, the detrimental effect of nicotine is no longer evident suggesting that detoxification costs might be counterbalanced by the advantages in slowing the progression of the infection. However, contrary to our prediction, we found no trade-off between costs and benefits in terms of survival, and infected bumblebee lifespan was not affected by the consumption on nicotine. Our analysis focussed on individual bumblebees in isolation and there might be many other subtle long term benefits we have not explored, such as possible benefits at the colony level. It might be that lifespan analysis of forager bumblebees would be different in a social setting. Infected bumblebees have impaired learning abilities ( Alghamdi *et al.*, 2008; Gegear *et al.*, 2006) and a reduction in parasite load might affect foraging efficiency or nursing ability, in turn enhancing colony productivity. Moreover, nicotine might be beneficial in slowing the progression of the infection through a colony, allowing, for example, the queen to lay more eggs or the larvae to prosper.

The cost imposed by the consumption of nicotine in our experiments may explain why healthy bees did not constantly consume high doses of nicotine ( Tiedeken *et al.*, 2014). Similarly, infected bees kept in Petri dishes reduced the overall uptake of food if exposed to nicotine. This is surprising given that those bumblebees also had a slight preference for sucrose solution laced with the alkaloid, and free-flying healthy bumblebees were not repelled by artificial nectar laced with nicotine. While these behavioural preferences may be explained by the impact that some nectar alkaloids, including nicotine, have on learning and memory in bees ( Chittka & Peng, 2013; Thany & Gauthier, 2005; Wright *et al.*, 2013), the mechanism behind the overall reduced consumption caused by nicotine remains unexplained. In humans at least, it is well established that nicotine has appetite-reducing effects ( Jessen *et al.*, 2005).

Currently it is unclear how nicotine acts on *C. bombi*. Nicotine is a highly toxic molecule ( Benowitz, 1998) that acts against a wide spectrum of bacterial and fungal pathogens ( Pavia *et al.*, 2000). House sparrows and several finch species, for example, add smoked cigarette butts retaining substantial amounts of nicotine to their nests to reduce mite infestations ( Suárez-Rodríguez *et al.*, 2013). While our *in vivo* microbiological experiments prove the antimicrobial activity of nicotine against the pathogen when ingested, they also suggest that nicotine does not directly interfere with the protozoan’s viability, at least when measured as infectivity. As suggested by Manson *et al.* (2010), who found similar effects of the natural alkaloid gelsemine, an alkaloid-rich diet might increase a bee’s excretion rate, as occurs for nectarivorous bird ( Tadmor-Melamed *et al.*, 2004), effectively “flushing” *C. bombi* cells from the gut. Another possibility might be that nicotine, or perhaps its metabolites, directly modify the mid-gut epithelium or the environment of its lumen, making it less suitable for the parasite.

In conclusion, we showed that when infected, bumblebees use a nectar alkaloid, slowing the progression of the infection. Nicotine consumption did not affect bee lifespan but the reduction in the parasite load may have other likely subtle benefits both for individual bees and colony. Recent findings confirm the suggestion that the preferential ingestion of natural nectar secondary metabolites in pollinators might play a key role in mediating pathogen transmission within and between colonies ( Richardson *et al.*, 2015) or interactions among pollinators and their parasites ( Manson *et al.*, 2010). The observed increased ingestion of a nectar alkaloid might be a generalized response to sickness and not just to *Crithidia*. Similarly, our results and other recent studies ( Gherman *et al.*, 2014; Richardson *et al.*, 2015) provide potential evidence for self-medication mediated by the consumption of plant secondary metabolites. Yet, the conditions under which nicotine and other alkaloids consumption provides benefits to either bees or plants remain to be identified. We thus believe that a careful approach to interpreting impacts of plant metabolites on insect parasites is warranted. The contention that secondary metabolites in nectar may be under selection from pollinators, or used by plants to enhance their own reproductive success ( Chittka & Peng, 2013; Thomson *et al.*, 2015; Wright *et al.*, 2013), should ideally be confirmed with further studies, which examine the impacts of these metabolites on both bee and plant fitness under field-realistic conditions.

### Data availability

The data referenced by this article are under copyright with the following copyright statement: Copyright: 2015 © Baracchi D et al

Data associated with the article are available under the terms of the Creative Commons Zero "No rights reserved" data waiver (CC0 1.0 Public domain dedication). 

F1000Research: Dataset 1. Infection experiments, 10.5256/f1000research.6262.d44610 ( Baracchi *et al.*, 2015a).

F1000Research: Dataset 2. Revision 1. Laboratory toxicity bioassays, 10.5256/f1000research.6262.d48008 ( Baracchi *et al.*, 2015b).

F1000Research: Dataset 3. Trade-off between detrimental and beneficial effects of nicotine, 10.5256/f1000research.6262.d44613 ( Baracchi *et al.*, 2015c).

F1000Research: Dataset 4. Diet preference of caged bees, 10.5256/f1000research.6262.d44614 ( Baracchi *et al.*, 2015d).

F1000Research: Dataset 5. Behavioural test, 10.5256/f1000research.6262.d44615 ( Baracchi *et al.*, 2015e).
