# Supplementary material for: Behavioural evidence for self-medication in bumblebees?
Source: F1000Res. 2015 Oct 29;4:73. Originally published 2015 Mar 19. [Version 3] doi: 10.12688/f1000research.6262.3 (PMC4406194; doi:10.12688/f1000research.6262.3)
Supplement: Peer review history

# Peer review: Referee response for version 2

**DOI:** 10.5256/f1000research.7009.r8767

**Published:** 2015-09-22

**Authors:** Michael Simone-Finstrom

**Affiliations:** 1 Department of Entomology, North Carolina State University, Raleigh, NC, USA

I have read this submission. I believe that I have an appropriate level of expertise to confirm that it is of an acceptable scientific standard.

# Peer review: Referee response for version 2

**DOI:** 10.5256/f1000research.7009.r9415

**Published:** 2015-07-08

**Authors:** James Thomson

**Affiliations:** 1 Department of Ecology and Evolutionary Biology, University of Toronto, Toronto, ON, Canada

I have now looked through the new version and can happily remove my reservations regarding the earlier version.  My main biological concern—that dosages of secondary metabolites need to be considered in the light of honeypot dynamics—is now given an intelligent discussion.  The problems with table entries and figure labels have also been corrected.  I did not see a need to change the title, but I agree that the new title is good and appropriate.

I have read this submission. I believe that I have an appropriate level of expertise to confirm that it is of an acceptable scientific standard.

# Peer review: Referee response for version 1

**DOI:** 10.5256/f1000research.6717.r8216

**Published:** 2015-04-20

**Authors:** Marla Spivak

**Affiliations:** 1 Department of Entomology, University of Minnesota, St Paul, MN, USA

Determining the extent that bumble bees may self-medicate by consuming floral nectar containing antimicrobial secondary plant metabolites when infected with a pathogen is a fascinating line of study.  The experiments presented are well conducted and analyzed, and I found the results not entirely surprising or contradictory. 

There was a temporary effect of nicotine (when provided in sugar syrup) against *Crithidia bombi* in *Bombus terrestris * workers. This effect might have been stronger and more long lasting, and even might have cleared the infection, if the dose of nicotine consumed was higher.  This could be tested in future experiments using the high- and low-end concentrations found in floral nectar.  Even though the infected bees preferred sucrose laced with nicotine in the clever foraging tests and in petri dishes, the authors say the results should be interpreted with caution because the life expectancy of the infected, nicotine-fed bees was not increased relative to controls.  But as they also point out, bees don’t live very long, and many factors affect worker bee life expectancy.  Possibly, at the colony level, reducing the infection level even for a few days in a certain number of workers might slow the rate of horizontal transmission among nest mates; this remains to be tested. 

I think it would be revealing to test the effects of these alkaloids, or other plant metabolites, on infected queen bumble bees. As *Crithidia *is also vertically transmitted, it would be interesting to know if the vertical transmission of this parasite could be reduced if the queen ingests nectar that contains antimicrobial metabolites.  It also would be interesting to know if the infected workers that collect nectar containing these compounds feed the queen with them, potentially lowering her pathogen load, which would then might allow her to produce more reproductive male and gynes.  These types of experiments are more difficult, but could yield more field-relevant results. 

Yet, the experiments presented here are great first steps in understanding a new area of research on the interaction of pollinators, pathogens and plant compounds.  I greatly appreciate that the authors offer a cautionary stance in interpreting their results, but I do think, given the physiological tradeoffs involved in consuming potentially toxic compounds, that small temporary effects on individual bees might translate to larger effects at the colony-level.

I have read this submission. I believe that I have an appropriate level of expertise to confirm that it is of an acceptable scientific standard.

# Response

**Published:** 2015-05-27

**Authors:** Lars Chittka

**Affiliations:** School of Biological and Chemical Sciences, Queen Mary, University of London, UK

Referee comment: Determining the extent that bumble bees may self-medicate by consuming floral nectar containing antimicrobial secondary plant metabolites when infected with a pathogen is a fascinating line of study.  The experiments presented are well conducted and analyzed, and I found the results not entirely surprising or contradictory. 

*Author reply: We agree both and we changed the title and the discussion accordingly.*

Referee comment: There was a temporary effect of nicotine (when provided in sugar syrup) against *Crithidia bombi* in *Bombus terrestris* workers. This effect might have been stronger and more long lasting, and even might have cleared the infection, if the dose of nicotine consumed was higher.  This could be tested in future experiments using the high- and low-end concentrations found in floral nectar.  Even though the infected bees preferred sucrose laced with nicotine in the clever foraging tests and in petri dishes, the authors say the results should be interpreted with caution because the life expectancy of the infected, nicotine-fed bees was not increased relative to controls.  But as they also point out, bees don’t live very long, and many factors affect worker bee life expectancy.  Possibly, at the colony level, reducing the infection level even for a few days in a certain number of workers might slow the rate of horizontal transmission among nest mates; this remains to be tested. 

I think it would be revealing to test the effects of these alkaloids, or other plant metabolites, on infected queen bumble bees. As *Crithidia *is also vertically transmitted, it would be interesting to know if the vertical transmission of this parasite could be reduced if the queen ingests nectar that contains antimicrobial metabolites.  It also would be interesting to know if the infected workers that collect nectar containing these compounds feed the queen with them, potentially lowering her pathogen load, which would then might allow her to produce more reproductive male and gynes.  These types of experiments are more difficult, but could yield more field-relevant results. Yet, the experiments presented here are great first steps in understanding a new area of research on the interaction of pollinators, pathogens and plant compounds.  I greatly appreciate that the authors offer a cautionary stance in interpreting their results, but I do think, given the physiological tradeoffs involved in consuming potentially toxic compounds, that small temporary effects on individual bees might translate to larger effects at the colony-level.

*Author reply: We agree and we now explain in the discussion that our results provide potential  evidence for self-medication. Yet, until measurable benefits for the bees or the colonies are found we think that caution is warranted.*

# Peer review: Referee response for version 1

**DOI:** 10.5256/f1000research.6717.r8215

**Published:** 2015-04-10

**Authors:** Michael Simone-Finstrom

**Affiliations:** 1 Department of Entomology, North Carolina State University, Raleigh, NC, USA

The authors presented a fairly comprehensive set of experiments in order to elucidate the role that nicotine may have in self-medication of bumblebees against *Chrithidia* infection. The experiments progress well, starting with controlled lab infection studies to lab-based diet and lifespan analysis to a semi-field foraging test. I think the authors sufficiently described the methods and subsequent results and mostly had an appropriate discussion of the relevant findings. I just have a few small issues that the authors could address to improve and clarify aspects of the manuscript. But overall it is a strong paper.

First, I do think “contradictory” could be removed from the title as I don’t think the results are necessarily contradictory, but do just show a weak effect. Just because the ingestion of nicotine reduces parasite infection and does not have a subsequent effect of increasing lifespan of infected bees, this does not mean that there are no other fitness-related benefits or that at different doses effects may not be more pronounced. I think “weak effect” aptly describes the findings.

Generally, tables and figures could be labeled more effectively. For example, I think the fact that Figure 1 and 2 have the same x-axis is problematic given what the experimental treatments were. I think in Figure 2 it would be better to indicate that the Nicotine there is the Nicotine pre-treatment. Same for Table 2. Whereas in Figure 1 it would be nicotine diet. The legend for Figure 3 should explicitly state that these bees were all uninfected, since this is an important point, and since the lifespan data of infected bees is not represented in a figure.

For the discussion and results, overall I think there just are a few other points that can be made. One is simply drawing stronger connections across the multitude of tests that were done.

Infection experiments: would it be valuable to show statistically that there is no difference between the pre-treated *Chrithidia *and the standard (basically comparing figure 1 and figure 2)? Also is the 2-hour exposure time relevant? How was this decided upon? Same for dose of exposure. I think this needs to be discussed more thoroughly or at least a citation provided to justify this amount.

Trade-off: Important to note that *Chrithidia *infection was found to reduce lifespan of bees (as compared to the bees in the toxicity part of the study).

I think a discussion of the relevance of the doses used is important. Were any dose-response trials conducted? Presumably at a higher dose, it would be even more toxic to the bees and lower doses may not have much of an effect.

Could ingestion of nectar alkaloids be a generalized response to sickness? So maybe it’s not as effective against *Chrithidia*, but this isn’t the only parasite bumblebees get.

A larger context into how this might influence colony dynamics and health is important. This is hinted at in a couple sentences, but since all of these studies were really done with individual bees or individual behavior, this is a significant point. Perhaps lifespan analysis of forager bumblebees would be different in a social setting. Similarly perhaps fitness benefits aren’t seen since it’s just measured in terms of individual lifespan, but maybe a reduction in parasite load affects foraging efficiency or nursing ability and thus colony productivity.

I have read this submission. I believe that I have an appropriate level of expertise to confirm that it is of an acceptable scientific standard.

# Response

**Published:** 2015-05-27

**Authors:** Lars Chittka

**Affiliations:** School of Biological and Chemical Sciences, Queen Mary, University of London, UK

Referee comment: First, I do think “contradictory” could be removed from the title as I don’t think the results are necessarily contradictory, but do just show a weak effect. Just because the ingestion of nicotine reduces parasite infection and does not have a subsequent effect of increasing lifespan of infected bees, this does not mean that there are no other fitness-related benefits or that at different doses effects may not be more pronounced. I think “weak effect” aptly describes the findings.

*Author reply: As suggested by the referee we removed the word “Contradictory” from the title and phrased it as a question instead.*

Referee comment: Generally, tables and figures could be labelled more effectively. For example, I think the fact that Figure 1 and 2 have the same x-axis is problematic given what the experimental treatments were. I think in Figure 2 it would be better to indicate that the Nicotine there is the Nicotine pre-treatment. Same for Table 2. Whereas in Figure 1 it would be nicotine diet.

*Author reply: As suggested by the referee we labelled more effectively the figures and the tables as outlined above.*

Referee comment: For the discussion and results, overall I think there just are a few other points that can be made. One is simply drawing stronger connections across the multitude of tests that were done.

*Author reply: We changed part of the discussion to address this concern.*

Referee comment: Infection experiments: would it be valuable to show statistically that there is no difference between the pre-treated *Crithidia *and the standard (basically comparing figure 1 and figure 2)?

*Author reply: As suggested by the referee we added the statistical test.*

Referee comment: Also is the 2-hour exposure time relevant? How was this decided upon? Same for dose of exposure. I think this needs to be discussed more thoroughly or at least a citation provided to justify this amount.

*Author reply: In the Materials and Methods section we provided a citation to justify the protocol used.*

Referee comment: Trade-off: Important to note that *Crithidia *infection was found to reduce lifespan of bees (as compared to the bees in the toxicity part of the study).

*Author reply: We stressed this point more clearly in the revised version.*

Referee comment: I think a discussion of the relevance of the doses used is important. Were any dose-response trials conducted? Presumably at a higher dose, it would be even more toxic to the bees and lower doses may not have much of an effect.

*Author reply: We agree and since the same point has been also raised by a second referee (James Thomson) we discuss carefully and more clearly this important point in the revised version.*

Referee comment: Could ingestion of nectar alkaloids be a generalized response to sickness? So maybe it’s not as effective against *Crithidia*, but this isn’t the only parasite bumblebees get.

*Author reply: The referee suggestion is valid and we now mention this in the discussion.*

Referee comment: A larger context into how this might influence colony dynamics and health is important. This is hinted at in a couple sentences, but since all of these studies were really done with individual bees or individual behavior, this is a significant point. Perhaps lifespan analysis of forager bumblebees would be different in a social setting. Similarly perhaps fitness benefits aren’t seen since it’s just measured in terms of individual lifespan, but maybe a reduction in parasite load affects foraging efficiency or nursing ability and thus colony productivity.

*Author reply: In line with the important comment, also raised by a second referee (James Thomson), we added a paragraph to further discuss these issues.*

# Peer review: Referee response for version 1

**DOI:** 10.5256/f1000research.6717.r8036

**Published:** 2015-03-27

**Authors:** James Thomson

**Affiliations:** 1 Department of Ecology and Evolutionary Biology, University of Toronto, Toronto, ON, Canada

**Content of paper**. This paper reports a set of nicely designed experiments aimed at determining whether *Bombus terrestris *self-medicates against trypanosome ( *Crithidia bombi) *infection by seeking and consuming nicotine-laced floral nectar. Using captive bee colonies and artificial flowers in laboratory conditions permits well-controlled manipulative experiments that would be effectively impossible in the field. The results are ambivalent, in that dietary nicotine does reduce the intensity of gut infection, and infected bees do seek out nicotine, but attempts to demonstrate improved health in terms of worker bee lifespan yield negative results.  For the most part, I endorse both the interpretation of these results and the recognition by Baracchi *et al.* that further research is needed to settle the question of self-medication. 

I do have three reservations about the interpretations offered here, however, and I think the paper would be improved by some additional discussion of these issues. First, any anti-parasitic medicinal effects of toxic compounds will depend on dose rates. Too much medicine may harm the host; too little may exert no therapeutic effect. Baracchi *et al.* state that they have used “natural” dosages of nicotine, but what that means is that they have prepared solutions whose nicotine levels match those reported from some floral nectars. That is an appropriate starting point, but there may be little correspondence between the concentrations that flowers offer and those that bees are exposed to in a natural colony. Foraging workers typically collect floral nectar from various plant species that differ in nectar chemistry. Much of what they collect is not digested by the collectors themselves, but is transported back to the nest and regurgitated into communal honeypots that serve as energy stores for the larvae, the queen, and the many workers that do not forage. As this complicated cocktail is assembled, soluble compounds are concentrated by evaporation, diluted by mixing with other nectars, and probably further modified by enzymatic and microbial action. There is no reason to expect that the concentration of any particular compound in this brew bears any relationship to its concentration in one of the many floral nectars that have been pooled. Indeed, honeypots within a nest may hold different mixtures because particular foragers tend to discharge their collections into particular honeypots. I believe that explicit attention must be paid to honeypot composition if the question of bee medication is to advance. 

Second, by choosing to look at toxic effects on workers, Baracchi *et al. *are not able to detect possible benefits of medication on other members of the colony, specifically larvae or the queen. One can imagine that certain inputs of nicotine to the colony might have no net effects of worker survival but might allow the queen to lay more eggs or the larvae to prosper. Indeed, effects could conceivably be harmful for the foragers but still beneficial to the colony. There might also be different effects on workers that forage and those that serve as nurses. The social nature of these bees must be considered. 

Third, by considering nectar only, Baracchi *et al.* don’t consider the probability that secondary metabolites found in nectar are also likely to occur in the pollen of the same flower species. In nature, therefore, bees that choose to forage on nicotine-rich nectars are also likely to be collecting nicotine rich pollen from those flowers. To the extent that such correlations hold in nature, the larvae (who are the primary consumers of that pollen) may be receiving very different doses than adult bees. 

In summary, it would be very illuminating, although tedious, to consider experiments that measure whole-colony health as a response variable rather than worker longevity.

**Errors.**  In this review process, the lack of line numbers, or an editable form of the MS, makes it hard to flag such things as unclear phrases.  Here are a few mistakes that should be addressed, however: 

In the abstract, the plant family *Solanaceae *is misspelled. 

In the caption for Figure 1, “bees received” should be rephrased. 

I believe that there must be a serious error in Dataset 2.  Unless I am missing something, the data reported for “starved” and “ *ad libitum*” treatments are identical. This looks like a cut-and-paste error. 

Column headings in the tables and data sets should be more explanatory. For example, in Table 2, experimental treatments are denoted simply as “time” and “nicotine.” Those labels are too cryptic. 

The "Statistical Analysis" section refers to several "classical...parameters," but these are not picked up in the Results. Reconcile?

I have read this submission. I believe that I have an appropriate level of expertise to confirm that it is of an acceptable scientific standard, however I have significant reservations, as outlined above.

# Response

**Published:** 2015-05-27

**Authors:** Lars Chittka

**Affiliations:** School of Biological and Chemical Sciences, Queen Mary, University of London, UK

Referee comment: …Any anti-parasitic medicinal effects of toxic compounds will depend on dose rates. Too much medicine may harm the host; too little may exert no therapeutic effect. Baracchi *et al.* state that they have used “natural” dosages of nicotine, but what that means is that they have prepared solutions whose nicotine levels match those reported from some floral nectars. That is an appropriate starting point, but there may be little correspondence between the concentrations that flowers offer and those that bees are exposed to in a natural colony. Foraging workers typically collect floral nectar from various plant species that differ in nectar chemistry. Much of what they collect is not digested by the collectors themselves, but is transported back to the nest and regurgitated into communal honeypots that serve as energy stores for the larvae, the queen, and the many workers that do not forage. As this complicated cocktail is assembled, soluble compounds are concentrated by evaporation, diluted by mixing with other nectars, and probably further modified by enzymatic and microbial action. There is no reason to expect that the concentration of any particular compound in this brew bears any relationship to its concentration in one of the many floral nectars that have been pooled. Indeed, honeypots within a nest may hold different mixtures because particular foragers tend to discharge their collections into particular honeypots. I believe that explicit attention must be paid to honeypot composition if the question of bee medication is to advance. 

*Author reply: We added a paragraph in the discussion section to address this point.*

Referee comment: By choosing to look at toxic effects on workers, Baracchi *et al. *are not able to detect possible benefits of medication on other members of the colony, specifically larvae or the queen. One can imagine that certain inputs of nicotine to the colony might have no net effects of worker survival but might allow the queen to lay more eggs or the larvae to prosper. Indeed, effects could conceivably be harmful for the foragers but still beneficial to the colony. There might also be different effects on workers that forage and those that serve as nurses. The social nature of these bees must be considered. 

*Author reply: We agree and we stressed more clearly in the revised version that benefits from the consumption of nicotine may* *influence colony health and dynamics despite the lack of evident benefits for infected foragers.*

Referee comment: By considering nectar only, Baracchi *et al.* don’t consider the probability that secondary metabolites found in nectar are also likely to occur in the pollen of the same flower species. In nature, therefore, bees that choose to forage on nicotine-rich nectars are also likely to be collecting nicotine rich pollen from those flowers. To the extent that such correlations hold in nature, the larvae may be receiving very different doses than adult bees. 

*Author reply: The argument raised by the referee is valid, and we briefly mentioned this possibility in the discussion*

Referee comment: In summary, it would be very illuminating, although tedious, to consider experiments that measure whole-colony health as a response variable rather than worker longevity.

*Author reply: We feel that considering a new set of experiments to measure whole-colony health would represent not simply an additional big experiment, but a new study itself.* *I* *nstead, we now carefully discuss all these issues.*

Referee comment: In the abstract, the plant family *Solanaceae *is misspelled. 

In the caption for Figure 1, “bees received” should be rephrased. 

I believe that there must be a serious error in Dataset 2.  Unless I am missing something, the data reported for “starved” and “ *ad libitum*” treatments are identical. This looks like a cut-and-paste error. 

*Author reply: Thank you for spotting these errors. In the revised version we have fixed them.*

Referee comment: Column headings in the tables and data sets should be more explanatory. For example, in Table 2, experimental treatments are denoted simply as “time” and “nicotine.” Those labels are too cryptic. 

*Author reply: As suggested by the referee we labelled more effectively the figures and the date sets*

Referee comment: The "Statistical Analysis" section refers to several "classical...parameters," but these are not picked up in the Results. Reconcile? 

*Author reply: In the revised version we have made sure that the Results section addressed all the parameters mentioned in the Methods section.*
